# How we diagnose and treat hereditary transthyretin-mediated amyloidosis with polyneuropathy in the Balkan region: an expert opinion

**DOI:** 10.3389/fneur.2026.1876381

**Published:** 2026-06-26

**Authors:** Ivailo Tournev, Janez Zidar, Borut Peterlin, Ervina Bilić, Stojan Perić, Sonja Pavlović, Teodora Chamova

**Affiliations:** 1 Clinic of Nervous Diseases at Aleksandrovska University Hospital; Department of Neurology, Medical University - Sofia, Sofia, Bulgaria; 2 Expert Center for Hereditary Neurologic and Metabolic Disorders Sofia, Sofia, Bulgaria; 3 Department of Cognitive Science and Psychology, New Bulgarian University Sofia, Sofia, Bulgaria; 4 Institute of Clinical Neurophysiology, Division of Neurology, University Medical Centre, Ljubljana, Slovenia; 5 Clinical Institute of Genomic Medicine Ljubljana, Ljubljana, Slovenia; 6 Department of Neurology Clinical Hospital Center Zagreb, University of Zagreb School of Medicine Zagreb, Zagreb, Croatia; 7 Faculty of Medicine, University of Belgrade, Belgrade, Serbia; 8 Laboratory for Molecular Hematology, Institute of Molecular Genetics and Genetic Engineering, University of Belgrade, Belgrade, Serbia

**Keywords:** Balkan region, disease management, expert opinion, hereditary transthyretin-mediated amyloidosis, patient journey

## Abstract

Hereditary transthyretin-mediated (ATTRV; v, variant) amyloidosis is a genetic disorder that causes abnormal accumulation of amyloid deposits in organs and tissues. The most common neurological manifestation is polyneuropathy (PN) of the autonomic nervous system, cardiac involvement and average survival of 6–12 years since onset of symptoms. Recent years have been marked by advancements in diagnosis and management of ATTRv amyloidosis with PN. In Balkan countries, endemic regions for some *TTR* gene mutations exist, with particularities in patient’s access to care given the different health infrastructure set-up. A panel of multidisciplinary experts from Bulgaria, Croatia, Serbia and Slovenia have gathered to discuss country-specific insights and approaches on the current ATTRv amyloidosis with PN diagnosis and management, sharing challenges and best practices. Each country has instituted various strategies for screening, genetic testing, treatment and follow-up of patients. As an endemic country with an older center of excellence in place, Bulgaria has currently a high rate (95%) of patients with ATTRv amyloidosis with PN diagnosed in the early stages of the disease. Across the other countries, delays in referral to a specialist for diagnosis and initiation of treatment have been noted due to insufficient awareness of the disease among other healthcare professionals, low availability of therapies or issues with reimbursement. Enhancement of interdisciplinary collaboration, clinical awareness and screening programs are the key areas of improvement in the Balkan region.

## 1 Introduction

Hereditary transthyretin-mediated (ATTRv; v, variant) amyloidosis (1) is a progressive disorder caused by mutations in the *TTR* gene leading to a systemic accumulation of misfolded TTR protein aggregates in different organs and tissues (2). This deposition largely manifests as sensorimotor polyneuropathy (PN) affecting small (sensory and autonomic) and large fibers, cardiomyopathy (CM), and/or gastrointestinal (GI) dysfunction (2). The estimated global prevalence of ATTRv amyloidosis in 2018 was of approximately 10,186 individuals (range: 5,526–38,468) (2). ATTRv amyloidosis is considered endemic in several European countries, including Bulgaria (1), Portugal, Sweden (3), Cyprus, and Spain (4).

ATTRv amyloidosis with PN is one of the most severe adult-onset hereditary neurodegenerative disorders, with a reduced survival from symptom onset (in average, ~6 to 12 years) (5). In non-endemic regions, diagnostic delays typically range from 3 to 4 years (5). Localized amyloid deposits can cause bilateral carpal tunnel syndrome (CTS), often years before a more widespread PN becomes apparent (2). To timely diagnose ATTRv amyloidosis with PN, clinicians should recognize the progression from distal sensory complaints to motor and autonomic involvement (2).

The aim of this paper is to present the current approaches to diagnosing and managing ATTRv amyloidosis with PN in four Balkan countries, each with their own healthcare setups. Analyzing clinical disparities at country level may optimize diagnostic pathways and enhance patient outcomes across the Balkan region.

## 2 Methods

A panel consisting of eight experts (neurologists, geneticists and radiologists) in neurologic rare disorders from Bulgaria, Croatia, Slovenia and Serbia met online in January 2025 to share their experience and country-specific insights on current ATTRv amyloidosis with PN management. This perspective is the result of discussions and answers provided to a questionnaire regarding diagnosis, treatment algorithms, patient pathway and care in each country.

## 3 Discussion

### 3.1 Diagnosis of ATTRv amyloidosis with PN

Bulgaria is considered an endemic country for ATTRv amyloidosis and has implemented a systematic screening program within affected families and rapid genetic testing, facilitating very early diagnosis in most cases. The age of onset varies largely (from 18 to over 75 years) because of different underlying genetic variants. A significant clinical heterogeneity in disease onset and severity, even among individuals with the same mutation has been noticed.

In Croatia, diagnosis is often more likely in families presenting predominant cardiac symptomatology. A common patient profile involves mild neuropathy that progresses, with cardiomyopathy (CM) the key trigger for diagnostic investigations.

In Serbia, all patients with a small fiber neuropathy or axonal sensory (motor) polyneuropathy of unknown cause are tested for ATTRv amyloidosis. Individuals presenting both CM and CTS are systematically referred from the cardiologist for neurological evaluation to investigate for potential ATTRv amyloidosis.

Slovenia implemented several strategies to allow an early diagnosis, including medical charts review of individuals previously diagnosed with idiopathic neuropathy and a systematic campaign to increase awareness on ATTRv amyloidosis diagnosis among different specialties (neurologists, cardiologists, hematologists, gastroenterologists).

#### 3.1.1 Genetic testing

Genetic testing of the *TTR* gene is fundamental to the diagnosis of ATTRv amyloidosis with PN and is routinely performed in all four countries for all patients with suspicion of disease. Sequence analysis of *TTR* successfully identifies approximately 99% of pathogenic variants, all of which are situated within exons 2, 3, and 4 (2). In patients presenting with unexplained progressive PN, even in the absence of other “red flag” symptoms suggestive of ATTRv amyloidosis, gene panel testing and/or laboratory screening can help exclude alternative causes of neuropathy, such as other genetic conditions, vitamin deficiencies, diabetes, or amyloid light-chain (AL) amyloidosis (6). Several mutations have already been identified in the Balkan region, Glu89Gln being the most common in Bulgaria and reported also in Slovenia. In Croatia, the most common is Asp38Glu and in Serbia one of the mutations reported is pVal50Met (Table 1).

**Table 1:** Identified *TTR* gene mutations in specific countries in Balkan region.

Country	Mutation	Occurence	Presentation
Bulgaria (1)	c.325G>C, p. Glu109>Gln (Glu89Gln) (18)	Endemic region; most common (107 affected families) – 73.48% of the carriers of mutations.	Present in patients with mean age of onset of 51 years and mixed phenotypes, involving the peripheral nerves, heart, and gastrointestinal system.
	c.148G>A, p. Val50Met (Val30Met) (18, 19)	Second most common (20 affected families) – 12.81% of the carriers of mutations.	Present in patients with mean age of onset of 64 years and mixed phenotypes and late onset.
	c.290C>T, p. Ser97Phe (Ser77Phe) (18)	Founder effect. All 14 families originate from small village Vakarel – 8.54% of the carriers of mutations.	Represented by a late-onset form (mean age at onset is 57 years) and in patients with mixed phenotype.
	c.200G>A, p. Gly67Glu (Gly47Glu) (18)	Roma families from North-Eastern part of Bulgaria – 1.80% of the carriers of mutations.	Present in patients with mean age of onset of 18–28 years and mixed phenotypes, involving the peripheral nerves, and gastrointestinal system.
	c.220_221GA>TT, p. Glu74Leu (Glu54Leu) (18, 20)	Three unrelated Bulgarian families – 1.80% of the carriers of mutations.	Present in patients with age at onset of 50 years and characterized by predominant cardiac involvement.
	c.214 T>C, p. Ser72Pro (Ser52Pro) (18)	One family – 0.22% of the carriers of mutations.	Present in patients with age at onset of 44 years and mixed phenotype.
	c.258A>C, p.Glu86Asp (Glu66Asp) (21)	One family – 0.45% of the carriers of mutations.	Present in patients with onset of disease of 55 years and characterized by predominant cardiac involvement (advanced amyloid cardiomyopathy) and minimal neurological involvement.
	c.238A>G, p.Thr80Ala, (Thr60Ala)	One family – 0.22% of the carriers of mutations.	Present in patients with onset of disease of 58 years and with mixed phenotype, but more severe cardiac involvement.
Croatia	c.114 T>A, p.Asp38Glu, (Asp18Glu)	The most common, endemic mutation for the south part of Croatia; reported in 20 patients.	Present in patients with asymptomatic or oligosymptomatic neuropathy in the third decade followed by cardiac symptoms.
	c.118G>A, p.Val40Ile, (Val20Ile)	Reported in one patient.	Present in patients with late onset CM.
	c.424G>A, p.Val142Ile, (Val122Ile)	Reported in one patient.	Present in patients with CM and subclinical sensory small fiber neuropathy.
Serbia	c.148G>A, p.Val50Met (Val30Met)	Two unrelated cases.	Present in one patient with neuropathy and CTS, followed by cardiac symptoms (CM and cardiac conduction blocks). Another patient with progressive sensorimotor polyneuropathy.
	c.133G>T, p.Ala45Ser, (Ala25Ser) Mutation already described in Sweden	One family.	Present in patients with mixed phenotypes, involving the peripheral nerves, heart (arrhythmias and CM), and gastrointestinal system (mostly diarrhea).
	c.251 T>C, p.Phe84Ser, (Phe64Ser)	One family.	Proband with polyneuropathy, uveitis, and the central nervous system involvement - leptomeningeal infiltration (depression, hemianopsia, hemiparesis, with later cognitive deterioration).
	c.424G>A, p.Val142Ile, (Val122Ile)	Common in African Americans; one case reported in Serbia.	Patient with autonomic impairment, mild sensory polyneuropathy, bilateral CTS, CM, and atrial fibrillation.
	c.325G>C, p.Glu109Gln (Glu89Gln)	Bulgarian and North Macedonian mutation; one case reported in Serbia.	Patient with progressive sensorimotor polyneuropathy and recurrent syncopes.
	c.176A>T, p.Asp59Val (Asp39Val)	One case reported in Serbia.	Patient with CM.
Slovenia	c.425 T>C, p.Val142Ala, (Val122Ala) (16)	Single family.	Patients with mixed phenotype.
	c.149 T>C, p.Val50Ala, (Val30Ala) (16)	Single family.	Patients with predominantly neuropathic phenotype.
	c.379A>T, p.Ile127Phe, (Ile107Phe)	Single family.	Patients with mixed phenotype.
	c.173A>C, p.Asp58Ala, (Asp38Ala)	Single family.	Patients with predominant cardiac phenotype.
	c.325G>C, p.Glu109Gln, (Glu89Gln)	Four families.	Patients with mixed phenotype.

Genetic testing is reported as readily accessible in Bulgaria, with results typically available within 3 days. Since the first family diagnosis in 2008, a proactive screening program was initiated in 2014, with 1,403 individuals tested, of which 445 were carriers of different mutations. This program targets 151 affected families to identify carrier status and subsequently monitor asymptomatic individuals, leading to the identification of over 170 asymptomatic carriers.

Genetic testing for ATTRv amyloidosis in Croatia started with a screening program involving 161 patients suspected of *TTR* mutation, with 22 found positive, counting three different mutations. The common causes of the neuropathic phenotype were ruled out before referral to genetic testing (Sanger sequencing). The most common mutation originates from Southern Croatia.

In Serbia, 335 patients with suspicion have been tested by sequencing *TTR* gene (exome sequencing), with 7 cases confirmed positive. Although the initial testing program ended in 2023, genetic testing is currently routinely performed through the clinical exome or whole exome sequencing funded by the Serbian Health Fund, as part of the diagnostic process for patients referred due to “red flags” suggestive of ATTRv amyloidosis. In Slovenia, genetic testing by exome sequencing has been initiated in 2013, with approximately 10–15 samples processed annually in patients with suspicion of disease.

#### 3.1.2 Clinical and paraclinical assessment

Typically, but not always, especially in non-endemic regions, the onset of ATTRv amyloidosis with PN is characterized by symptoms indicative of small fiber neuropathy. This usually manifests with positive and negative sensory testing symptoms, as painful paresthesia and numbness in the lower extremities with autonomic impairment (2).

Nerve conduction study (NCS) is instrumental in the diagnosis of patients with suspicion of ATTRv amyloidosis with PN by objectively characterizing the nature of the peripheral large-fiber neuropathy particularly in non-endemic regions or in cases without a known family history (5). In Bulgaria and Serbia, NCS is routinely performed. In Bulgaria, it is performed in *TTR* variant carriers, irrespective of the presence of symptoms (7).

Sympathetic skin response (SSR) measures transient changes in skin electrical potential, primarily generated by sweat gland (sudomotor) activity (8). In Bulgaria, SSR is routinely performed in carriers, irrespective of the presence of symptoms and is positive very early in symptomatic carriers, as observed by physicians (7).

Sudoscan assesses autonomic small nerve fiber impairment by measuring electrochemical skin conductance (ESC) (9). It shows considerable promise as a reliable marker for detecting early dysautonomia, a common feature of ATTRv amyloidosis with PN (9). In Bulgaria, Sudoscan is routinely performed in carriers, irrespective of the presence of symptoms. It is used to diagnose the transition from asymptomatic carrier to symptomatic case and thereafter, to monitor the effect of treatment (7). In Croatia, it is the first exam performed as part of the diagnostic process in all patients with a suspicion of sensory small fiber neuropathy.

Biopsy is not commonly included in the current protocols for ATTRv amyloidosis diagnosis, while it may be performed to confirm the presence of amyloid deposits (6). In Croatia, skin biopsy is available for all patients with suspicion of ATTRv amyloidosis with PN. In Slovenia, skin and other tissue biopsy with staining for amyloid detection is used to help diagnose small fiber neuropathy.

Serum neurofilament light chain (sNfL) is becoming a valuable biomarker in neurological disorders (10), as sNfL levels have been shown to correlate with both clinical and electrophysiological measures of disease severity (10). In the context of endemic ATTRv amyloidosis in Bulgaria, sNfL assessment is used as an early indicator of neuropathy and to monitor treatment efficacy.

According to the Bulgarian clinical expertise, both SSR and Sudoscan can be affected by factors such as temperature and psychological state of the patient. For increasing sensitivity and specificity, the experts recommend combining Sudoscan with SSR or NCS.

#### 3.1.3 “Red flags” for diagnosing ATTRv amyloidosis with PN

Most frequently reported “red flags” in patients presenting with symptoms of neuropathy are early autonomic disfunctions (i.e., erectile dysfunction, light-headedness from postural hypotension, changes in bowel movement misdiagnosed as irritable bowel syndrome), and lack of response for neuropathy-specific treatments (i.e., steroids, intravenous immunoglobulin or plasma exchange for chronic inflammatory demyelinating polyneuropathy) (6). In addition, experts from Croatia, Slovenia and Serbia agreed that the rapid progression rate of PN is a common “red flag.”

### 3.2 From asymptomatic carrier to symptomatic patient

**Figure 1:**
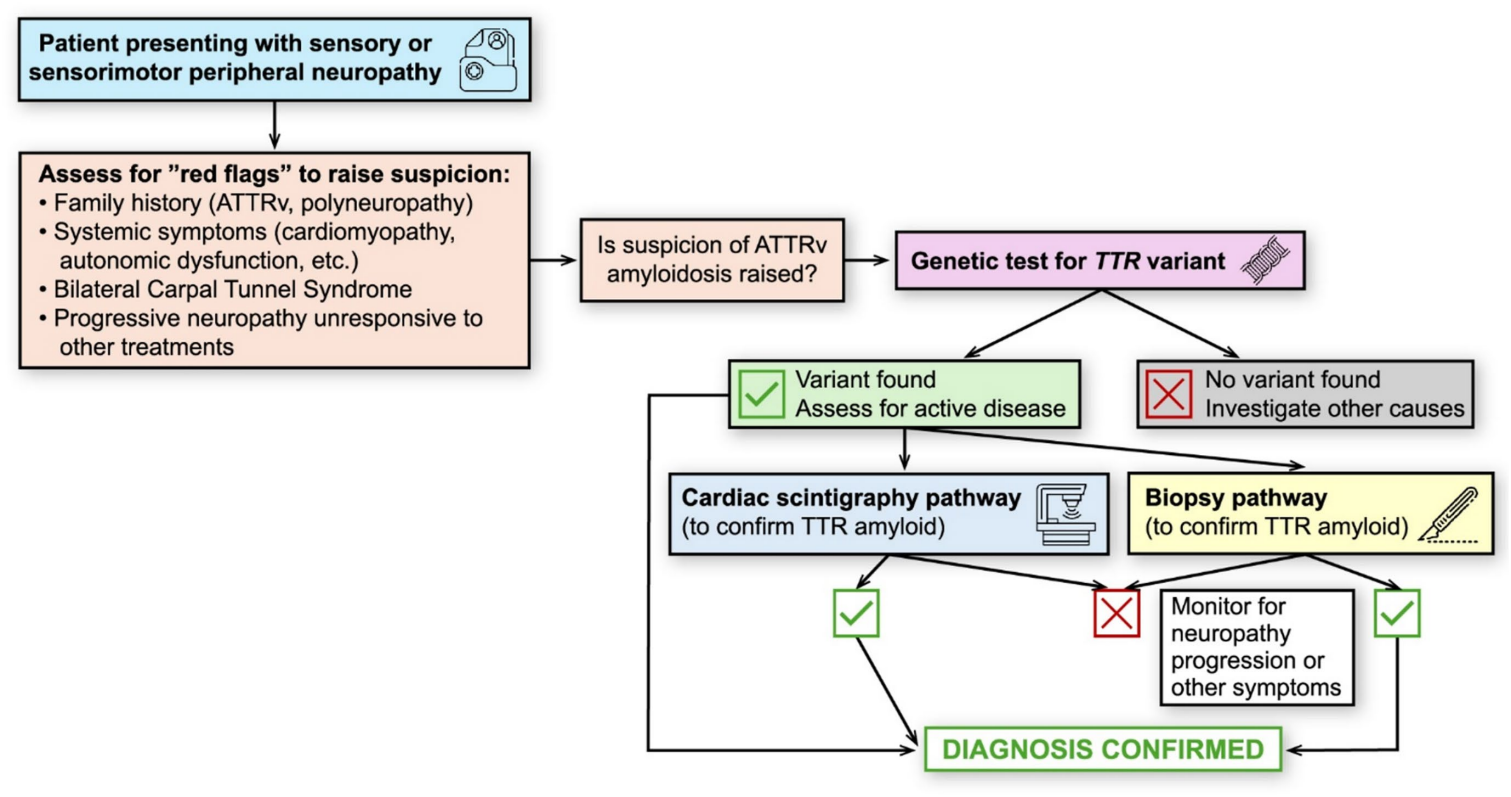
Diagnostic process of patients with ATTRv amyloidosis with PN. ATTRv amyloidosis, hereditary transthyretin-mediated amyloidosis; ATTRv amyloidosis with PN, hereditary transthyretin-mediated amyloidosis with polyneuropathy; TTR, transthyretin.

Notably, not all individuals carrying a *TTR* gene variant will have a clinical expression of ATTRv amyloidosis, and the identification of a *TTR* variant does not necessarily indicate active disease (6). A guided diagnostic process based on experts’ opinions from Balkan countries is presented in Figure 1.

In Bulgaria, depending on initial symptoms, the patient’s first medical encounter is either with GP or with a neurologist, cardiologist, gastroenterologist, and then referred to the Expert ATTRv Center in University Hospital Aleksandrovska from Sofia, that acts as Center of Excellence, established in 2016. All patients and asymptomatic carriers are monitored by a multidisciplinary team (MDT) every 6 months.

In Croatia, the first medical visit for ATTRv amyloidosis with PN may be at their GP or physical therapist and referred to the neurologist to establish the diagnosis (clinical exam, electromyoneurography, quantitative sensory testing, bloodwork, and autonomic testing). Genetic testing is performed at the Department for Personalized Medicine in the Clinical Hospital Center Zagreb.

In Serbia, patients initially present at the GP, neurologist, cardiologist, gastroenterologist or even at psychiatrist and referred to neurology for advanced testing (NCS and quantitative sensory testing) and diagnosis. Genetic testing is accessible at the Institute of Molecular Genetics and Genetic Engineering in Belgrade and is covered by the National Insurance Fund. Cardiac scintigraphy and heart MRI are also frequently part of the diagnostic tests for all suspected patients.

In Slovenia, a similar patient journey is identified, with the first medical encounter being with GP, neurologist, cardiologist or gastroenterologist, then referred to the Center of Excellence in Ljubljana. Families from Macedonia (endemic region) with a positive family history and CTS, receive early diagnosis and treatment. In other cases, delays to the correct diagnosis have been noted.

### 3.3 Treatment

The treatment strategy in ATTRv amyloidosis with PN focuses on: (i) suppression of amyloidogenic TTR synthesis, use of TTR silencers (eplontersen, inotersen, patisiran, vutrisiran) and liver transplantation to eliminate circulating mutant TTR protein, (ii) stabilization of TTR tetramers to prevent misfolding, with TTR stabilizers (tafamidis, diflunisal), and (iii) removal of existing TTR amyloid fibrils, with TTR disrupters (anti-TTR monoclonal antibodies) (11).

The treatment algorithm in Bulgaria follows the European Consensus for diagnosis, management, and treatment of transthyretin familial amyloid polyneuropathy (4): first a TTR stabilizer, that could continue for long-term in patients diagnosed early with stable disease, without any neurologic progression. Progression of disease is assessed using the neuropathy impairment score in the lower limb (NIS-LL) (12). An increase of at least 9 points suggests switching from tafamidis 20 mg to TTR silencers. The most important factors in deciding to initiate TTR stabilizers or TTR silencers are severity of symptoms and clinical presentation, genetic confirmation of ATTRv amyloidosis, and availability of specific treatments (13).

Eplontersen, inotersen, patisiran, tafamidis and vutrisiran are all available and reimbursed by The National Health Insurance Fund in Bulgaria, based on a protocol issued by The Expert Center for Hereditary Neurologic and Metabolic Disorders. Tafamidis is reimbursed for both dosage forms, tafamidis meglumine 20 mg (for ATTRv amyloidosis with PN) and tafamidis 61 mg (for ATTRv amyloidosis with CM). In Croatia, all five treatments are available, and only vutrisiran is currently reimbursed. TTR stabilizers are rarely used here for managing ATTRv amyloidosis with PN. Even with a proper diagnosis, drug availability could be challenging.

In Serbia, tafamidis is available and reimbursed, and vutrisiran is approved and reimbursed for three diagnosed patients. Therapies in Serbia are funded by the specific Fund for Rare Disease. Therapies are to be approved by the specific commission of the Fund for Rare Diseases. The treatment for ATTRv amyloidosis with PN is usually tafamidis, which is currently available in tertiary centers. In Slovenia, all five treatments are available, but eplontersen is not currently reimbursed. The treatment starts with TTR stabilizers (tafamidis) as soon as ATTRv amyloidosis with PN is diagnosed.

Taking together, in all countries, treatment for ATTRv amyloidosis with PN is initiated with tafamidis. The most common barrier to timely diagnosis and treatment of ATTRv amyloidosis with PN in countries from Balkan region is the lack of awareness among healthcare professionals. Some other barriers reported include high cost of treatment options, as seen in Serbia and Croatia.

### 3.4 Follow-up and long-term management

Clinicians should first optimize all symptomatic treatments to distinguish true disease progression from treatable mimics (6). Experts agreed that regular follow-up is recommended every 6 months and should involve a neurologic examination and focused history to track disease progression and screen for complications, including the development or worsening of cardiac issues (6).

As follow-up, the patients in Bulgaria undergo neurological examinations combined with NCS, Sudoscan and SSR for evaluating the small fibers, since small fiber neuropathy can be present about one year before the nerve conduction studies show changes. In Bulgaria and Serbia, a typical follow-up frequency for ATTRv amyloidosis with PN patients is ensured every 6 months, and in Croatia and Slovenia every 3 to 6 months.

#### 3.4.1 Multidisciplinary care approach

Experts iterated the need for increased clinical awareness, especially among cardiologists, neurologists, gastroenterologists, nephrologists, neuro-ophthalmologists, and physiotherapists, in order to establish a timely diagnosis.

In Bulgaria, the strategy for the diagnosis and management of ATTRv amyloidosis is based on the work of the Center of Excellence. According to the study by Nakov et al. (2021) (14), this approach has led to the identification of a significant number of new symptomatic patients, and over 100 asymptomatic carriers of the mutation, who are now included in a structured monitoring program. With this strategy, approximately 95% of patients in Bulgaria are diagnosed at early stages of the disease (14).

In recent years, the diagnostic approach to ATTRv amyloidosis in Croatia has seen substantial progress. A key development was the launch of the Croatian Transthyretin Cardiac Amyloidosis (CroATTR) Registry in September 2022, which laid the groundwork for a more organized and proactive strategy. All cases of ATTRv amyloidosis with PN in Croatia are discussed on regular basis on Neuromuscular Multidisciplinary Team Meetings (15).

In Serbia, cardiologists play a key role in the referral of patients with suspected amyloidosis to a neurologist specialized in PNs and to the medical institute for genetic testing and confirmation of diagnosis.

The proactive diagnostic approach currently seen in Slovenia has developed notably over the past decade. As early as 2015, reports pointed to increasing awareness among neurologists and cardiologists as a key driver in identifying new cases (16). At that time, only a small number of affected families had been recognized, but the foundation for a more systematic strategy was already being established. This shift was largely motivated by the recognition of the varied clinical presentations of the disease, ranging from mixed neuropathic-cardiomyopathic forms linked to the Val122Ala mutation to predominantly neuropathic forms associated with the Val30Ala mutation (16).

### 3.5 Patient education

Genetic counseling should precede genetic testing for individuals with ATTRv amyloidosis suspicion. If a pathogenic *TTR* variant is identified, counseling and testing should be extended to at-risk family members who may also be carriers (6). In Bulgaria from 2013 to 2025, ATTRv amyloidosis multidisciplinary experts’ team have participated in 13 national annual meetings of the patient organization “FAP Bulgaria,” providing education about diagnostics, treatment and follow-up of the disease to patients and asymptomatic carriers.

In Croatia, information regarding the disease is provided to both patients and their families by MDT members. Croatia has a strong tradition and ongoing development of programs dedicated to rare diseases, with regular activities such as the International Rare Disease Day, the National Rare Disease Day, and similar initiatives.

In Slovenia, a digital family history tool is planned for use as a general screening method in the primary healthcare sector. Approximately 50% of individuals could be at risk based on family history, highlighting thus how important it is to comprehensively collect family history. In contrast, in Serbia, many cases are sporadic with negative family history and variable disease expression within families, complicating identification.

To improve diagnostic rates and clinical outcomes in the Balkan region, experts suggested diverse educational and systemic strategies, including:

- Development of tailored materials emphasizing phenotypic variability and the importance of genetic counseling.
- Implementation of standardized family history tools and accessible information for patients.
- Advanced training for clinicians in genetic risk communication and referral optimization.
- Active partnership with patient organizations to bridge awareness gaps.

### 3.6 Physician education

In Bulgaria, since 2012, ATTRv amyloidosis has been extensively discussed at national congresses and professional meetings across multiple disciplines involved in the diagnosis and monitoring of the disease, including neurology, cardiology, gastroenterology, and GP. These educational activities have raised awareness among diverse medical communities and provided a platform for presenting and promoting the national screening program implemented in Bulgaria.

In Croatia, over the past 5 years, three dedicated workshops for neurology residents have been organized, covering topics such as *Small Fiber Neuropathies*, *ATTRv amyloidosis with Polyneuropathy – How to Shorten the Journey Toward Diagnosis*, and *Neuropathy as a Rare Disease*. National neurological conferences also covered these topics, and educational materials focusing on the diagnosis and treatment of ATTRv amyloidosis with PN have been published. Recommendations for diagnostics and treatment of ATTRv amyloidosis with PN are published in a manual entitled “*Guidelines for diagnostics and treatment of neuromuscular diseases”* by authors Bilić Ervina and Borovečki Fran (17).

In Serbia, a regional meeting with international guest lecturers, called *Neuropathy Update* is being organized every 4 years. At all meetings from 2014 to 2018 and to 2022, TTR was one of the topics included. Next meeting will take place in 2026, and it will focus on complex neuropathy phenotypes affecting other organs besides peripheral nerves, including TTR.

In Bulgaria, the therapeutic guidelines are revised periodically (available in Bulgarian and English) and distributed by the Bulgarian Society of Neurology to all neurologists (18). National ATTRv amyloidosis guidelines are not available in other Balkan countries.

## 4 Conclusion

ATTRv amyloidosis with PN management in the Balkans faces shared challenges, including diagnostic delays and the need for enhanced professional education. Key “red flags”—specifically CTS and CM alongside small-fiber neuropathy—must trigger immediate referral to centralized expert centers, to prevent fragmented diagnostic journeys. Regional cooperation is vital to balance best practices and ensure equitable access to care.

Treatment should be initiated as soon as symptomatic disease is confirmed. Asymptomatic carriers from affected families in which a disease-causing mutation has been identified through genetic testing should undergo regular monitoring to enable prompt detection of disease onset. Treatment should follow a stepwise protocol: initiating stabilizers (e.g., tafamidis) with a clear escalation pathway to gene silencers when disease progresses. Expanding access to TTR silencers in Serbia and Croatia is a critical regional priority. Finally, long-term management requires lifelong, multidisciplinary follow-up to monitor therapeutic efficacy and clinical progression.

## Data Availability

The original contributions presented in the study are included in the article/supplementary material, further inquiries can be directed to the corresponding author.
